# A replication-defective Japanese encephalitis virus (JEV) vaccine candidate with NS1 deletion confers dual protection against JEV and West Nile virus in mice

**DOI:** 10.1038/s41541-020-00220-4

**Published:** 2020-08-05

**Authors:** Na Li, Zhe-Rui Zhang, Ya-Nan Zhang, Jing Liu, Cheng-Lin Deng, Pei-Yong Shi, Zhi-Ming Yuan, Han-Qing Ye, Bo Zhang

**Affiliations:** 1 Key Laboratory of Special Pathogens and Biosafety, Wuhan Institute of Virology, Center for Biosafety Mega-Science, Chinese Academy of Sciences, Wuhan, China; 2 University of Chinese Academy of Sciences, 100049 Beijing, China; 3 Department of Biochemistry and Molecular Biology, University of Texas Medical Branch, Galveston, TX 77555 USA; 4 Drug Discovery Center for Infectious Disease, Nankai University, 300350 Tianjin, China

**Keywords:** Vaccines, Live attenuated vaccines

## Abstract

In our previous study, we have demonstrated in the context of WNV-ΔNS1 vaccine (a replication-defective West Nile virus (WNV) lacking NS1) that the NS1 *trans*-complementation system may offer a promising platform for the development of safe and efficient flavivirus vaccines only requiring one dose. Here, we produced high titer (10^7^ IU/ml) replication-defective Japanese encephalitis virus (JEV) with NS1 deletion (JEV-ΔNS1) in the BHK-21 cell line stably expressing NS1 (BHK_NS1_) using the same strategy. JEV-ΔNS1 appeared safe with a remarkable genetic stability and high degrees of attenuation of in vivo neuroinvasiveness and neurovirulence. Meanwhile, it was demonstrated to be highly immunogenic in mice after a single dose, providing similar degrees of protection to SA14-14-2 vaccine (a most widely used live attenuated JEV vaccine), with healthy condition, undetectable viremia and gradually rising body weight. Importantly, we also found JEV-ΔNS1 induced robust cross-protective immune responses against the challenge of heterologous West Nile virus (WNV), another important member in the same JEV serocomplex, accounting for up to 80% survival rate following a single dose of immunization relative to mock-vaccinated mice. These results not only support the identification of the NS1-deleted flavivirus vaccines with a satisfied balance between safety and efficacy, but also demonstrate the potential of the JEV-ΔNS1 as an alternative vaccine candidate against both JEV and WNV challenge.

## Introduction

The *Flavivirus* genus of the *Flaviviridae* family consists of many important human arboviral pathogens responsible for encephalitis and hemorrhagic diseases, such as Japanese encephalitis virus (JEV), West Nile virus (WNV), Dengue virus (DENV), yellow fever virus (YFV), Zika virus (ZIKV), tick-borne encephalitis virus (TBEV), St. Louis encephalitis viruses (SLEV)^1^. Among these, JEV is the leading cause of viral encephalitis in the Asia-Pacific area, causing nearly 68,000 cases of Japanese encephalitis (JE) each year with the case fatality rates averaged around 30%. Even those who survive JE (~50%) often suffer from permanent neuronal disorders like cognitive, motor, and behavioral impairments^2–5^. No effective antiviral therapeutics against JEV is available.

JEV vaccines are therefore the only effective approach to prevent JEV infection. The currently used JEV vaccines are classified into four main types: inactivated mouse brain-derived vaccines, inactivated Vero cell-derived vaccines, live attenuated vaccines, and live chimeric vaccines^6^. The inactivated JEV vaccines derived either from mouse brain^7^ or Vero cells^8,9^ are relatively safer but require repeated doses to achieve adequate protection. For the live attenuated/chimeric vaccines, only one-dose administration is enough to induce protective immunity against JEV infection. SA14-14-2 and ChimeriVax-JE are the two most widely used live attenuated/chimeric vaccines. SA14-14-2, an attenuated strain derived from its wild-type (WT) JEV SA14 strain^10,11^, is generated through multiple passages of SA14 virus in primary hamster kidney (PHK) cells and in mouse brain/non-neural tissues plus repeated plaque purifications^11^. ChimeriVax-JE is a live recombinant vaccine by replacement of the genes encoding two structural proteins (preMemebrane (prM) and Envelope (E)) of a YFV vaccine strain (YFV-17D) with the corresponding genes of JEV SA14-14-2 strain^12–14^. This chimeric virus replicates like YFV-17D, but elicits specific immunity against the heterologous JEV surface antigens. Despite the excellent safety record of SA14-14-2, the concern about the potential virulence reversion remains^10,15,16^.

Recently, we generated a replication-defective WNV-ΔNS1 vaccine candidate with a deletion of viral nonstructural protein 1 (NS1) by utilizing the complementing cell line expressing NS1 protein. This WNV-ΔNS1 exhibited high levels of safety and efficacy in mice^17^. In this study, we extend the NS1 trans-complementary platform to the development of JEV vaccines. The high titers of replication-defective JEV-ΔNS1 viruses with an NS1 deletion were produced using the previously established BHK-21 stable cell line that expresses WT WNV NS1 protein (BHK_NS1_). Through in vitro blind passage in BHK_NS1_ cells and in vivo neuroinvasiveness and neurovirulence evaluation, we demonstrated that JEV-ΔNS1 was genetically stable and highly attenuated. Meanwhile, the results of vaccine efficacy showed that a single dose of JEV-ΔNS1 vaccine could protect C57BL/6 mice from a highly lethal challenge with WT JEV. Importantly, we also found JEV-ΔNS1 induced cross-protective immune responses against the challenge of heterologous WNV, another important member in the same JEV serocomplex, accounting for up to 80% survival rate following a single dose of immunization relative to mock-vaccinated mice. Our study indicates the potential of the JEV-ΔNS1 as an alternative safe and effective vaccine candidate against both JEV and WNV infection.

## Results

### Generation and characterization of JEV-ΔNS1 particles

**Fig. 1:**
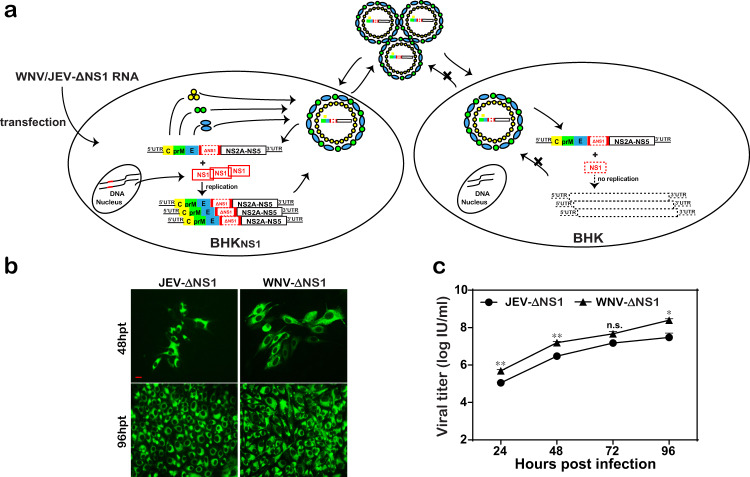
High replication efficiency of JEV-ΔNS1 in BHK_NS1_ cell line. **a** Schematic diagram of the generation and replication of JEV-ΔNS1 particles in cells. JEV-ΔNS1 (with a deletion of the residues 4–298 in NS1 coding sequence) replicates efficiently in the BHK-21 cell line stably expressing WNV-NS1 protein (BHK_NS1_), while undergoes a single round of entry, release and viral RNA translation in the normal cells. **b** IFA detection of JEV-ΔNS1 and WNV-ΔNS1 in BHK_NS1_ cells post transfection. Equal amounts of JEV-ΔNS1 and WNV-ΔNS1 RNAs were transfected into BHK_NS1_ cells. IFA analysis using 4G2 monoclonal antibody was performed at the indicated time points. The length of the scale bar (displayed in a red line segment) represents 20 μm. **c** Comparison of growth kinetics of JEV-ΔNS1 and WNV-ΔNS1. BHK_NS1_ cells were infected with JEV-ΔNS1 and WNV-ΔNS1 virus at an MOI of 0.1. The supernatants were harvested at the indicated time points and viral titers were determined by IFA on BHK_NS1_ cells as described in “Methods”. Two independent experiments were performed in triplicate. Data represent the mean ± standard deviation (SD) of the triplicate measurements in a representative experiment. Statistical analysis was performed with unpaired *t* test and the asterisks denote statistical differences between the indicated groups. *p < 0.05; **p < 0.01; n.s. no statistical difference.

Previously, we reported that WNV-ΔNS1 could replicate in Vero_NS1_ cell line efficiently^17^. In the present study, using the same method, we generated JEV-ΔNS1 particles through transfection of the transcribed JEV-ΔNS1 RNA into BHK_NS1_ cells stably expressing WNV NS1 protein (Fig. 1a). JEV-ΔNS1 particles replicated efficiently in BHK_NS1_ cells (Fig. 1b) with viral titers as high as 1 × 10^7^ IU/ml at 96 h post infection (hpi) (Fig. 1c).

**Fig. 2:**
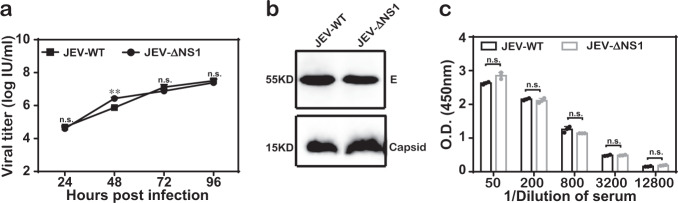
Characterization of JEV-ΔNS1. **a** Comparison of growth kinetics between WT JEV (JEV-WT) and JEV-ΔNS1 virus in BHK_NS1_ cells. BHK_NS1_ cells were infected with either JEV-WT or JEV-ΔNS1 virus at an MOI of 0.1. The supernatants were harvested at the indicated time points and viral titers were determined as described above. Data represent the mean ± standard deviation (SD) of the triplicate measurements in a representative experiment. Statistical analysis was performed with unpaired *t* test and the asterisks denote statistical differences between the indicated groups. **p < 0.01; n.s. no statistical difference. **b** Western blotting analysis of purified JEV-WT and JEV-ΔNS1 viral particles. Following concentration and purification through PEG8000 precipitation and ultracentrifugation, equal amounts of purified WT and ΔNS1 viruses were subjected to Western blotting analysis using the specific anti-E monoclonal antibody or anti-C polyclonal antibody. All blots derived from the same experiment and were processed in parallel. **c** ELISA for the detection of the antibodies against both JEV and JEV-ΔNS1. Two independent experiments were performed in triplicate. Data represent the mean ± standard deviation (SD) of the triplicate measurements in a representative experiment. Statistical analysis was performed with unpaired *t* test. n.s. no statistical difference.

Then, we compared viral growth kinetics, viral proteins in virions and viral antigenicity between WT JEV (JEV-WT) and JEV-ΔNS1. (i) Similar viral growth curves were observed in BHK_NS1_ cells for these two viruses at a multiplicity of infection (MOI) of 0.1, with the highest titers of about 1 × 10^7^ IU/ml at 96 hpi (Fig. 2a). (ii) After concentration and purification through PEG8000 precipitation and ultracentrifugation, the purified virions were subjected to western blotting analysis using envelope- (E) and capsid-specific (C) antibodies. Similar amount of viral proteins were detected in JEV-WT and JEV-ΔNS1 virion samples. (iii) The enzyme-linked immunosorbent assay (ELISA) was performed to detect the viral antigenicity using either JEV-WT or JEV-ΔNS1-coated 96-well plates. No significant differences in antigenicity between JEV-WT and JEV-ΔNS1 were observed as the same dilution of antisera against JEV-WT could equally recognize JEV-ΔNS1 antigen, suggesting that JEV-ΔNS1 virion remains antigenically intact (Fig. 2c).

### Genetic stability of JEV-ΔNS1 particles

**Fig. 3:**
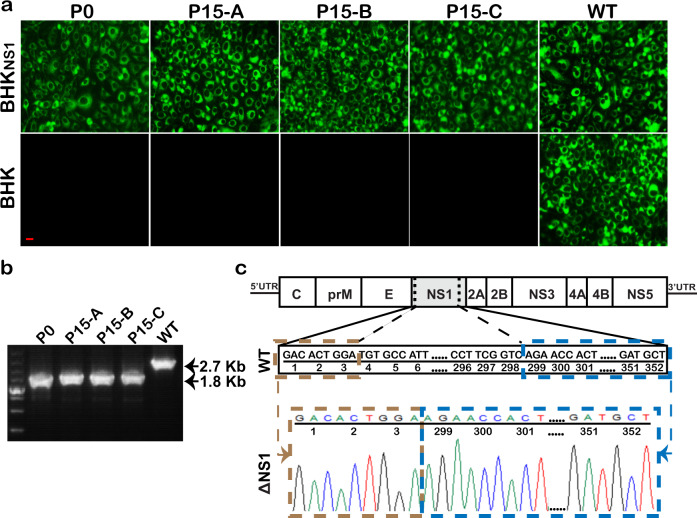
Stability of JEV-ΔNS1. **a** IFA analysis of JEV-ΔNS1 virus at different passages using 4G2 monoclonal antibody. The JEV-ΔNS1 virus generated through transfection of BHK_NS1_ cells with the transcribed JEV-ΔNS1 genomic RNA was designated as P0 virus. Three JEV-ΔNS1 virus stocks (A, B and C) were blind passaged independently for 15 rounds (P0−P15) in BHK_NS1_ cells. The viruses at P0 and P15 were used to infect BHK_NS1_ cells and naïve BHK-21 cells. JEV-WT was used as a positive control. The length of the scale bar (displayed in a red line segment) represents 20 μm. **b** RT-PCR analysis of the expression of NS1 gene in BHK_NS1_ cells infected with P0 or P15 JEV-ΔNS1 viruses. The uncropped and unprocessed gel including markers was displayed in Supplementary Fig. 2. **c** Sequence chromatograms of RT-PCR products containing NS1 fragment amplified from P15 JEV-ΔNS1 virus.

To investigate the stability of this vaccine candidate, three groups of JEV-ΔNS1 particles (Groups A, B and C) were passaged serially, independently, in BHK_NS1_ cells for 15 rounds (P0−P15). JEV-ΔNS1 viruses at P0 and P15 (P15-A, -B and -C) were used to infect BHK_NS1_ and the parental BHK-21 cells. All the infected cells were subjected to IFA and RT-PCR assay. As shown in Fig. 3a, the BHK_NS1_ cells infected with either group of P15 JEV-ΔNS1 produced robust IFA-positive signals, almost equivalent to those infected with P0 JEV-ΔNS1 or JEV-WT. In contrast, no IFA-positive signals were observed in all viruses except WT-infected BHK-21 cells. RT-PCR assay was carried out using the primer pair spanning the region from E to the C-terminus of NS2B that covers the complete NS1 coding sequence. The expected 2.7- and 1.8-kb RT-PCR products were detected in WT and JEV-ΔNS1 samples, respectively (Fig. 3b). Meanwhile, the sequencing result of NS1 coding region of P15 JEV-ΔNS1 also provided consistent evidence that the passaged virus still retained the same 295-residue deletion (residues 4-298) in NS1 protein (Fig. 3c). Overall, we confirmed that JEV-ΔNS1 virus is genetically stable and did not recombine with the WT NS1 sequences in the BHK_NS1_ cells.

### Assessment of neuroinvasiveness and neurovirulence of JEV-ΔNS1 in mice

**Fig. 4:**
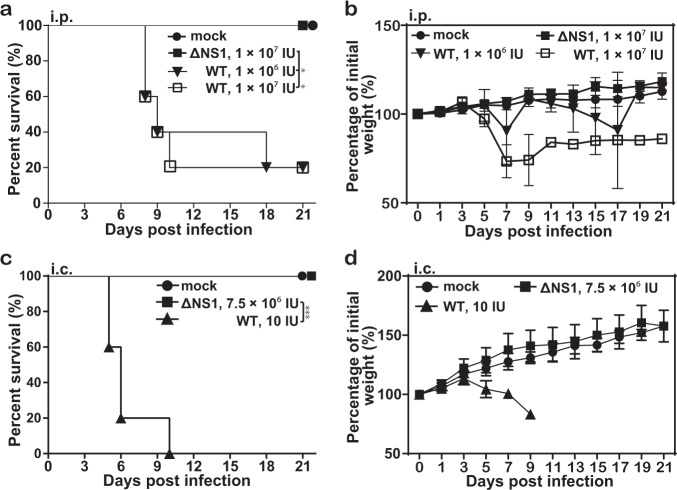
JEV-ΔNS1 is highly safe in mice. **a**, **b** Neuroinvasiveness tests for WT and JEV-ΔNS1 viruses in mice. Four-week-old C57BL/6 female mice (*n* = 5 for each group) were i.p. inoculated with 10^6^ and 10^7^ IU of WT JEV or 10^7^ IU of JEV-ΔNS1. The mortality (**a**) and weight loss (**b**) were monitored for 21 days. **c**, **d** Neurovirulence tests for WT and JEV-ΔNS1 viruses in mice. Three-week-old ICR female mice (*n* = 5 for each group) were i.c. inoculated with 10 IU of WT JEV or 7.5 × 10^6^ IU of JEV-ΔNS1. The mortality (**c**) and weight loss (**d**) were monitored for 21 days. Kaplan−Meier survival curves were analyzed by the log-rank test and the asterisks denote statistical differences between the indicated groups. *p < 0.05; ***p < 0.001. Two independent experiments were performed, and data from one experiment are presented.

To further evaluate the biosafety of JEV-ΔNS1 as vaccine candidate, we compared the neuroinvasiveness of the WT and JEV-ΔNS1 viruses in mice. Groups of 4-week-old C57BL/6 mice were inoculated intraperitoneally (i.p.) with 10^5^, 10^6^ and 10^7^ IU of WT JEV or 10^7^ IU of JEV-ΔNS1; the mortality of the infected mice was monitored for 3 weeks. In both WT virus-infected groups, almost all the mice showed serious diseases, such as ruffled fur and weight loss since day 5 post infection (Fig. 4b), and only one mouse in each WT group survived at the end of the observation period (Fig. 4a). In JEV-ΔNS1 virus-infected group, however, all the mice survived (Fig. 4a) with steadily increasing body weight and no signs of illness (Fig. 4b). We also compared the neurovirulence of the WT and JEV-ΔNS1 viruses in mice. Three-week-old ICR mice were inoculated intracranially (i.c.) with 10 IU of WT or 7.5 × 10^6^ IU of JEV-ΔNS1. As shown in Fig. 4c, d, all the WT-inoculated mice died within 10 days (Fig. 4c) and displayed symptoms (ruffled fur, hunched back, paralysis) from day 3 post infection (Fig. 4d). In spite of inoculation with 100,000-fold higher dosage of JEV-ΔNS1 viruses, all the mice survived without neurological symptoms (Fig. 4c, d). Collectively, these results indicate that the JEV-ΔNS1 virus is highly safe in mice.

### Protective efficacy of JEV-ΔNS1 against virulent JEV challenge in C57BL/6 mice

**Fig. 5:**
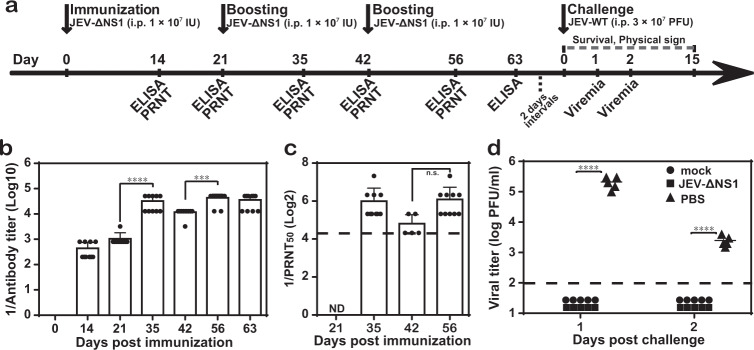
Protection of JEV-ΔNS1 in mice against JEV challenge. **a** Schematic diagram of animal experiment design and schedule. Four-week-old C57BL/6 female mice (*n* = 10 for each group) were i.p. immunized three times at 3-week intervals with 1 × 10^7^ IU of JEV-ΔNS1. At the indicated time points, the sera harvested from immunized mice were subjected to ELISA (**b**) and PRNT_50_ (**c**) to determine the IgG antibody and neutralizing antibody titers against JEV, respectively. ND not detected. **d** Viremia levels in WT JEV challenged mice. All mice in each group were i.p. injected with 3 × 10^7^ PFU of WT JEV at 23 days after the third immunization. The serum viremia levels were quantified by plaque assay in BHK-21 cells on days 1 and 2 post challenge. Mice without any treatment (mock) were used as a negative control. The dashed lines in panels (**b**−**d**) represent the limits of detection. Data represent the mean ± standard deviation of ten mice at each time point in each group. Statistical analysis was performed with one-way ANOVA and the asterisks denote statistical differences between the indicated time points in panels (**b**) and (**c**) or different groups in panel (**d**). ***p < 0.001; ****p < 0.0001; n.s. no statistical differences.

To evaluate the potency of JEV-ΔNS1 as a vaccine candidate, we firstly examined the antibody response induced by JEV-ΔNS1 in immunocompetent C57BL/6 mice. Four-week-old C57BL/6 mice (*n* = 10 for each group) were i.p. immunized three times at 3-week intervals. Each time the mice were inoculated with 1 × 10^7^ IU of JEV-ΔNS1 (Fig. 5a). As shown in Fig. 5b, JEV-ΔNS1 immunization induced robust antibody titers assayed by ELISA on day 14 (1:440) and day 21 (1:1040), and the following boosters further elevated the antibody titers to 1:43,520 on day 56 and afterwards (Fig. 5b). In agreement with the ELISA titers, neutralizing antibody titers reached 1:68 after the third immunization despite lower than the limit of our detection after the primary immunization (Fig. 5c). The results demonstrate that JEV-ΔNS1 could induce a robust antibody response.

To determine the protective efficacy of JEV-ΔNS1 virus against WT JEV, all the mice immunized with either phosphate-buffered saline (PBS) or JEV-ΔNS1 were i.p. challenged with 3 × 10^7^ PFU of WT JEV 23 days after the third immunization (Fig. 5a). As expected, all the JEV-ΔNS1-immunized mice remained healthy without any signs of illness during the observation period of 15 days. In contrast, the PBS-immunized mice started to show signs of disease (such as ruffled fur and paralysis) from day 6 post challenge. But none of them succumbed to infection, which may attribute to, as previously reported^18–20^, the age-dependent resistance to JEV infection. The viremia levels were measured on days 1 and 2 post challenge. All the mice immunized with PBS developed high levels of viremia, with the average viremia titers of about 10^5^ PFU/ml and 10^3^ PFU/ml on days 1 and 2 post challenge, respectively, in contrast to undetectable viremia in the JEV-ΔNS1-immunized group (Fig. 5d). Taken together, these results show that JEV-ΔNS1 could protect mice from virulent challenge of WT JEV.

### Protective efficacy of single immunization with JEV-ΔNS1 in C57BL/6 mice

**Fig. 6:**
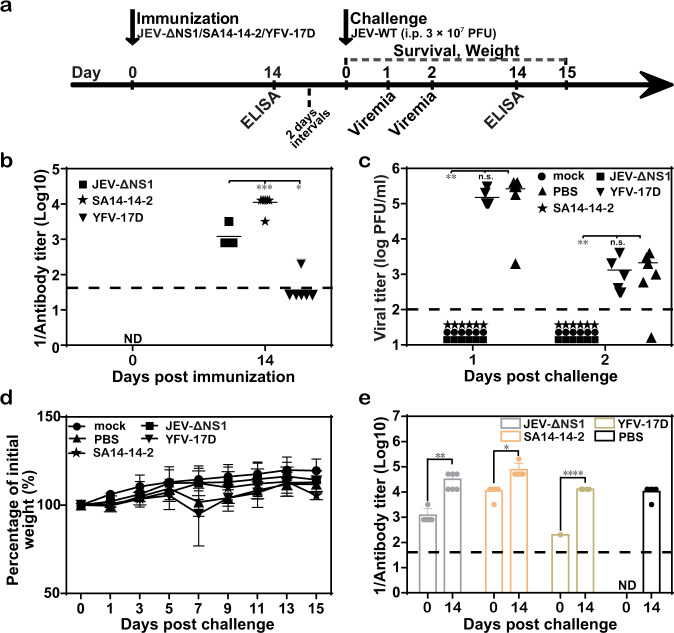
Protective efficacy of one-shot immunization with JEV-ΔNS1 in C57BL/6 mice. **a** Schematic diagram of animal experiment design and schedule. Four-week-old female C57BL/6 mice (*n* = 6 for each group) were i.p. immunized once with 1.4 × 10^7^ IU of JEV-ΔNS1 and SA14-14-2 or 6 × 10^5^ IU of YFV-17D. At 14 days after the immunization, the sera harvested from immunized mice was subjected to ELISA to detect the IgG antibody titers against JEV (**b**). ND not detected. **c**, **d** Viremia levels and weight changes in WT JEV challenged mice. All mice were i.p. injected with 3 × 10^7^ PFU of WT JEV at 16 days post immunization and monitored for viremia (**c**), weight loss (**d**), and IgG post-challenge titers (**e**). The dashed lines in panels (**b**, **c** and **e**) represent the limits of detection. Data represent the mean ± standard deviation of six mice at each time point in each group. Statistical analysis was performed with unpaired *t* test and the asterisks denote statistical differences between the indicated groups. *p < 0.05; **p < 0.01; ***p < 0.001; ****p < 0.0001; n.s. no statistical differences. Two independent experiments were performed, and data from one experiment are presented.

**Fig. 7:**
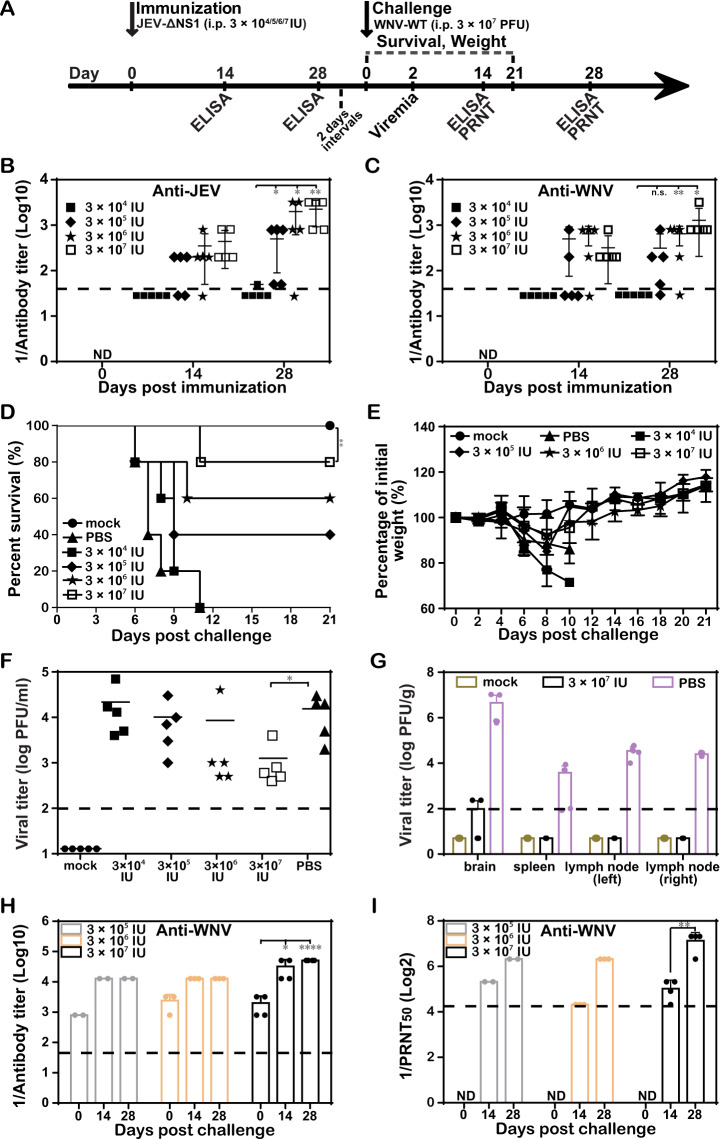
Cross-protection against WNV with one-shot low-dose immunization of JEV-ΔNS1. **a** Schematic diagram of animal experiment design and schedule. **b**, **c** ELISA for the detection of antibody titers. Four-week-old female C57BL/6 mice (*n* = 10 for PBS- and 3 × 10^7^ IU of JEV-ΔNS1-immunized groups; *n* = 5 for the other groups) were i.p. immunized once with different dosages of JEV-ΔNS1 (3 × 10^4^, 3 × 10^5^, 3 × 10^6^, or 3 × 10^7^ IU). On days 14 and 28, the sera were collected for the measurement of IgG antibody titers against JEV (**b**) and WNV (**c**), respectively. All mice were i.p. injected with 3 × 10^7^ PFU of WT WNV at 30 days post immunization. The mortality (**d**) and weight loss (**e**) were monitored daily. The viremia (**f**), viral load in various organs (**g**), IgG post challenge titers (**h**) and neutralizing antibody titers (**i**) against WNV at the indicated times post challenge were assayed as described in “Methods”. The dashed lines in panels (**b**, **c**, **f**−**i**) represent the limits of detection. Data represent the mean ± standard deviation (SD) of five mice at each time point in each group. Statistical analysis was performed with unpaired *t* test and Kaplan−Meier survival curves were analyzed by the log-rank test. The asterisks denote statistical differences between the indicated groups. *p < 0.05; **p < 0.01; ****p < 0.0001; n.s. no statistical differences.

To further explore whether a single vaccination with JEV-ΔNS1 was sufficient to elicit protective immunity against JEV, C57BL/6 mice were immunized only once with JEV-ΔNS1 prior to challenge. We also compared the vaccine efficacy of JEV-ΔNS1 with the commonly used JEV live attenuated vaccine, SA14-14-2 (the JEV live attenuated vaccine) at the same dosage. At the same time, the commercial YFV-17D vaccine (the YFV live attenuated vaccine) was used as a negative control. Sera collected on day 14 were subjected to ELISA (Fig. 6a). As shown in Fig. 6b, on day 14, the average IgG titers against JEV were 1:1200 and 1:11,200 for the groups treated with JEV-ΔNS1 and SA14-14-2, respectively; and only weak IgG titers were detected in one mouse of the YFV-17D immunized group. At 16 days post immunization, all mice, including PBS-vaccinated ones, were i.p. challenged with 3 × 10^7^ PFU of WT JEV. Mice were then monitored daily for weight loss and visible signs of disease. All mice received either JEV-ΔNS1 or SA14-14-2 vaccine remained healthy, with undetectable viremia (Fig. 6c) and gradually rising body weight (Fig. 6d), while the PBS- and YFV-17D-vaccinated mice exhibited high levels of viremia with the average viremia titers of >10^5^ and >10^3^ PFU/ml on days 1 and 2 post challenge, respectively (Fig. 6c), and started to display disease symptoms like weight loss from day 5 after the infection (Fig. 7d) (though recovered gradually from day 7). Also, the IgG antibody titers against JEV were measured at 0 and 14 days post challenge. The titers increased significantly in mice immunized with different vaccines, relative to their counterparts prior to challenge (Fig. 6e). Altogether, our results suggest that a single dose of JEV-ΔNS1 vaccine was able to confer efficient protection against JEV, which was equivalent to the licensed JEV vaccine.

### Cross-protection against WNV with one-shot low-dose immunization of JEV-ΔNS1

To further exploit the vaccine potential of JEV-ΔNS1, heterologous protection against WNV was investigated. The C57BL/6 mice were vaccinated once with four dosages (3 × 10^4^, 3 × 10^5^, 3 × 10^6^ and 3 × 10^7^ IU) of JEV-ΔNS1, respectively. ELISA assays were performed on days 14 and 28 post immunization for the titration of JEV- and WNV-reactive antibodies in mouse sera (Fig. 7a). It showed that JEV-ΔNS1 vaccination induced high levels IgG antibodies against both JEV and WNV in a dose-dependent manner. Specifically, the average anti-JEV titers were 1:50, 1:500, 1:2000, 1:2240 and the average anti-WNV titers were undetectable, 1:312, 1:650 and 1:1280 in the sera of mice immunized with 3 × 10^4^, 3 × 10^5^, 3 × 10^6^ and 3 × 10^7^ IU of JEV-ΔNS1 vaccines at 28 days post vaccination, respectively (Fig. 7b, c). At 30 days post immunization, all mice were i.p. challenged with 3 × 10^7^ PFU of WT WNV and observed daily for weight change and disease. Consistent with the cross-reactive IgG response to WNV, immunization of JEV-ΔNS1 also provided a dose-dependent protection against virulent WNV challenge. Except the lowest dosage (3 × 10^4^ IU), vaccination with 3 × 10^5^, 3 × 10^6^ and 3 × 10^7^ IU of JEV-ΔNS1 enhanced the survival rate to 40%, 60% and 80%, respectively, in comparison with the PBS-vaccinated group (Fig. 7d). In addition, these three groups of mice exhibited increasing body weight (Fig. 7e) during the whole observation period, and low levels of viremia at 2 days post challenge (Fig. 7f). On day 4 post challenge, the viral loads in a variety of organs of mice immunized with PBS (Fig. 7g) were also detected. In contrast to high levels of viral loads in all the tested organs of PBS-vaccinated mice, the JEV-ΔNS1-vaccinated mice only had a minor level of viruses in the brain (Fig. 7g). The IgG antibody and neutralizing antibody titers against WNV were also increased in mice immunized with different dosages of JEV-ΔNS1 relative to the counterparts prior to challenge (Fig. 7h, i). Overall, our results illustrated that JEV-ΔNS1 could protect mice from a highly lethal challenge of WNV.

## Discussion

Currently, there have been various inactivated and live attenuated/chimeric JEV vaccines available^7,12,15^, but the concerns about the efficacy or safety greatly limit their wide utilization. Flavivirus NS1 is a highly conserved glycoprotein with multiple functions in different forms^21^. Intracellular NS1 dimer is associated with the viral replication complex on the surface of the endoplasmic reticulum membrane and plays an essential role in viral RNA replication through modulating different viral and host factors^22–25^. The replication defect caused by NS1 mutations could be restored in *trans* by ectopic expression of homologous or even heterologous NS1 protein. Secreted and cell surfaced-associated NS1 are highly immunogenic and contribute to viral pathogenesis and immune evasion^26–30^. Antibodies against NS1 have been demonstrated to be protective against flavivirus infection. NS1-based subunit and DNA vaccines against JEV, DENV, YFV and ZIKV are developed at different stages^21,31^. In this study, we develop the replication-defective vaccine candidate, JEV-ΔNS1, using the NS1 *trans*-complementary platform we previously established^17^. It shows that JEV-ΔNS1 combines the safety and efficacy advantages of inactivated and live attenuated/recombinant vaccines: (i) It efficiently replicates in NS1 *trans*-complementing cell line but not in normal cells (Figs. 1 and 2a, b), and displays satisfied safety with good genetic stability (Fig. 3) and no apparent neurovirulence and neuroinvasiveness phenotypes in mice inoculated with high doses of JEV-ΔNS1 (Fig. 4); (ii) it maintains intact surface antigens that could not only be equally recognized by WT JEV antisera in ELISA assay (Fig. 2c), but also stimulate immune responses for efficient protection of mice against WT JEV challenge (Fig. 6).

In the vaccine efficacy assay, a commercial live attenuated JEV vaccine, SA14-14-2, was also used as a positive control. Vaccination with equal infectious titers of JEV-ΔNS1 gave similar degrees of protection to that of SA14-14-2, with healthy condition, undetectable viremia (Fig. 6c) and gradually rising body weight (Fig. 6d), although induced ~10-fold lower IgG antibody titers than vaccination with SA14-14-2. The differences in antibody titers between these two vaccines may result from their different replication capabilities: unlike SA14-14-2 continuously replicating in vivo, JEV-ΔNS1 only undergoes a single round of entry, release and viral antigens translation due to the NS1 deletion-caused complete block of viral genome replication.

Remarkably, a robust heterologous protection against WNV was observed in mice immunized with one-shot of JEV-ΔNS1 (Fig. 7). Both JEV and WNV belong to the antigenically related JEV serocomplex of flaviviruses. WNV is commonly found in Africa, Europe, the Middle East, North America and West Asia^32–34^. Recently, there have been reported clinically IgM-positive cases and isolated WNV from mosquito pools in China where JEV is endemic^34,35^. For the viruses without vaccines like WNV, cross-protective vaccination represents a promising approach against antigenically related viruses^36–38^. The cross-reactivity and protection of JEV vaccines against the closely related WNV have been investigated by different groups^1,39–41^. It showed that the natural infection of JEV or the vaccination of live attenuated/chimeric vaccines (SA14-2-8/ChimeriVax-JE) usually afforded strong cross-protective immunity against WNV infection^38–41^, with an exception that vaccination of SA14-14-2 failed to induce WNV-reactive antibodies in human recipients^36,42^. In comparison, immunization with inactivated vaccines provided robust heterologous protection against lethal WNV challenge only when co-delivery with YFV-17D vaccine^43^ or administration with Advax adjuvant^44,45^. It may attribute to the broad-based immune arsenal recruited by the adjuvants^45^. In the current study, JEV-ΔNS1 vaccine functions like live or attenuated/chimeric viruses, providing robust cross-protection of immunized mice against lethal WNV challenge with one-shot administration. Due to the sensitivity of PRNT assay we used here, the neutralizing antibody was not detectable after one-dose immunization despite the robust induction of antibody titers observed in ELISA assay. Nevertheless, we do believe that neutralizing antibody could be induced. As shown in Fig. 5c, after two rounds of vaccination, the neutralizing antibodies could be detected. Consistently, JEV-ΔNS1 vaccination induced strong cross-reactive antibody against WNV with an average titer 1:1280 at the highest dosage, only slightly less than the titer of anti-JEV antibody (1:2240) (Fig. 7).

In brief, our results showed that JEV-ΔNS1 is a safe and effective vaccine candidate, providing strong dual protection against both JEV and WNV challenge, and serving as a powerful tool to prevent both viruses infection, especially in their co-circulating areas.

## Methods

### Cells, viruses and antibodies

BHK-21 cells (ATCC® CCL-10™) were grown inDulbecco’s modified Eagle’s medium (DMEM) (Gibco) containing 10% heat-inactivated fetal bovine serum (Gibco), 100 units/ml of penicillin, 100 µg/ml of streptomycin in 5% CO_2_ at 37 °C. The stable BHK-21 cell line expressing NS1 protein (named as BHK_NS1_) was established following the same protocol as described previously for the generation of Vero_NS1_^17,46^. BHK_NS1_ cells were grown in DMEM as described above plus 0.8 µg/ml of puromycin (Invitrogen) in 5% CO_2_ at 37 °C. It is worthwhile noting that Vero cells are more widely accepted for vaccine study. Unfortunately, we lost our Vero cell line stably expressing WNV NS1 protein (Vero_NS1_) used in our previous study^17^. Alternatively, we conducted our study using another pre-established BHK_NS1_ cell line. The selection of a new Vero_NS1_ cell line is also undergoing for our future study. The reason we did not use JEV NS1 for packaging cells is that we had already obtained WNV NS1 expressing cell line and found that WNV NS1 could *trans*-complement JEV-ΔNS1 efficiently. Additionally, we hypothesize that NS1 from different virus may further decrease the possibility of recombination to produce WT virus. Recombinant WT JEV and WT WNV were generated by electroporation of BHK-21 cells with the transcribed viral genomic RNA from the linearized infectious cDNA clone of SA-14^47^ and strain 3356 from New York City^48,49^, respectively. JEV-ΔNS1 vector was constructed in the context of WT JEV cDNA clone following the same method as described previously for the generation of WNV-ΔNS1^17,46^, and the resultant replication-defective JEV-ΔNS1 and WNV-ΔNS1 viruses were obtained by lipofection (DMRIE-C Reagent, Invitrogen) of BHK_NS1_ cells with the transcribed viral genomic RNA from the linearized infectious cDNA clone of JEV-ΔNS1 and WNV-ΔNS1, respectively. The supernatants of transfected cells were harvested at 96 h post transfection (hpt) and frozen aliquots at −80 °C.

Monoclonal antibody 4G2 against the flavivirus envelope protein was kindly provided by Cheng-Feng Qin (Beijing Institute of Microbiology and Epidemiology, Beijing, China). Anti-JEV capsid polyclonal antibody was purchased from Abcam (China). Fluorescein isothiocyanate (FITC)-conjugated goat anti-mouse IgG and horseradish peroxidase (HRP)-conjugated goat anti-mouse/rabbit IgG secondary antibodies were purchased from Proteintech (China).

### Mice

Four-week-old C57BL/6 mice were used for virus neuroinvasiveness, immunization and challenge studies, and 3-week-old ICR mice were used to test the neurovirulence of JEV-ΔNS1. All mice infection experiments related to JEV were performed at an animal biosafety level 2 (ABSL-2) facility in Wuhan Institute of Virology under a protocol approved by the Laboratory Animal Ethics Committee of Wuhan Institute of Virology, CAS (Permit number: WIVA26201902). All mice infection experiments related to WNV were performed at an animal biosafety level 3 (ABSL-3) facility in Wuhan Institute of Virology under a protocol approved by the Laboratory Animal Ethics Committee of Wuhan Institute of Virology, CAS (Permit number: WIVA26201801).

### Vaccine strains

SA14-14-2 (the JEV live attenuated vaccine strain) was kindly provided by Prof. Geng-Fu Xiao (Wuhan Institute of Virology, Chinese Academy of Sciences, Wuhan, China). YFV-17D (the YFV live attenuated vaccine) was purchased from Beijing Tiantan Biological Products Co., Ltd.

### Viral titers quantification with immunofluorescence assay (IFA)

BHK_NS1_ cells seeded on coverslips in 12-well plates (2 × 10^5^ BHK_NS1_ cells per well) were infected with tenfold dilutions of JEV, JEV-ΔNS1 or WNV-ΔNS1 for 1 h at 37 °C before being overlaid with 2% methylcellulose. At 48 hpi, the infected cells were washed three times with PBS, followed by fixation by cold 5% acetone in methanol for 15 min at room temperature. Then, the fixed cells were stained with 4G2 antibody for 1 h at room temperature, washed three times with PBS, and incubated with FITC-conjugated goat anti-mouse IgG polyclonal secondary antibody. After three washes with PBS, the coverslips were mounted on glass slides with 90% glycerol. Images were captured under a fluorescence microscope (Nikon Eclipse TE2000). The infectious titer of viruses was calculated as IU per milliliter.

### Virus growth kinetics

The virus growth kinetics of JEV, JEV-ΔNS1 and WNV-ΔNS1 were performed in BHK_NS1_ cells. The cells were infected with viruses at a density of 2 × 10^5^ cells/well in 12-well plates (MOI = 0.1). At the indicated time points post infection, the supernatants were collected and subjected to viral titer quantification as described above.

### Concentration and purification of viral particles

The concentration and purification of WT JEV or JEV-ΔNS1 viral particles were carried out as described previously except that the supernatants were collected at 96 hpi^17^. Briefly, the supernatants reclaimed from infected BHK_NS1_ cells were subjected to sequential centrifugation to obtain the clarified stocks. Following incubation overnight at 4 °C with PBS containing 8% (wt/vol) PEG8000 (Sigma), the samples were centrifugated at 4 °C for 50 min at 10,500 × *g*. After gently resuspended with PBS, the pellets were subjected to ultracentrifugation at 4 °C for 1.5 h at 105,000 × *g* with a 24% sucrose cushion, using a SW41 rotor in an Optima MAX-XP ultracentrifuge (Beckman). The pellets were finally resuspended in 50 μl of PBS.

### Blind passage of JEV-ΔNS1 in BHK_NS1_ cells

The JEV-ΔNS1 derived from BHK_NS1_ cells transfected with in vitro transcribed JEV-ΔNS1 genomic RNA was designated as P0 virus for the following blind passage. Three independent passages (A, B and C) were performed. Initially, BHK_NS1_ cells growing in cell culture dish (35 mm) were infected with JEV-ΔNS1 (P0) at an MOI of 0.01, and at 3 days post infection, the supernatants were collected as P1. All the subsequent passages were carried out by repeatedly infecting naïve BHK_NS1_ cells in a 35 mm culture dish with 10 μl of culture medium from the previous passage and incubating for 3−5 days.

### Gel electrophoresis

After being heated at 95 °C for 10 min in SDS sample buffer containing β-mercaptoethanol, the denatured virion samples were subjected to electrophoresis on a 15% SDS-PAGE. The gel was then subjected to western blotting analysis as described previously^46^. All blots derived from the same experiment and were processed in parallel, the blots we presented in Fig. 2b were cropped into proper view and the raw data including molecular weight markers were displayed in Supplementary Fig. 1.

### Mouse neuroinvasiveness and neurovirulence tests

For neuroinvasiveness test, 4-week-old female C57BL/6 mice were infected i.p. with 10^6^ and 10^7^ IU of WT JEV or 10^7^ IU of JEV-ΔNS1. For neurovirulence test, 3-week-old female ICR mice were infected i.c. with 10 IU of WT JEV or 7.5 × 10^6^ IU of JEV-ΔNS1. The animals were all observed for survival and body weight changes for 21 days.

### Immunity and challenge studies

Four-week-old female C57BL/6 mice were i.p. immunized three times at 3-week intervals with 1 × 10^7^ IU of JEV-ΔNS1; the mice immunized with PBS were used as a negative control. At 23 days after the third vaccination, all the mice were i.p. challenged with 3 × 10^7^ PFU of WT JEV and monitored for survival and physical sign for 15 days. For one-shot immunization, three cohorts of mice were i.p. immunized once with 1.4 × 10^7^ IU of JEV-ΔNS1 and SA14-14-2 and 6 × 10^5^ IU of YFV-17D, respectively, followed by i.p. challenge with 3 × 10^7^ PFU of WT JEV at 16 days post immunization. The weight loss and survival were monitored for 15 days. For heterologous protection assay, the mice were i.p. immunized once with 3 × 10^4^, 3 × 10^5^, 3 × 10^6^ or 3 × 10^7^ IU of JEV-ΔNS1, then i.p. challenged with 3 × 10^7^ PFU of WT WNV at 30 days post immunization and monitored for weight loss and survival for 21 days. Viremia was quantified at 1 day and 2 days post challenge by plaque assay in BHK-21 cells as described previously^48,50^, and the limit of detection was 10^2^ PFU/ml. The IgG antibody titers were also quantified on days 14 and 28 after challenge.

### Organ virus titers

Organs from half of PBS and the highest JEV-ΔNS1 vaccine dosage (3 × 10^7^ IU) treated mice were removed, homogenized, and measured for viral loads on day 4 post challenge by plaque assay in BHK-21 cells as described above.

### Enzyme-linked immunosorbent assay (ELISA)

ELISA was used to measure antigenicity of JEV-ΔNS1 and JEV-specific IgG antibody levels of vaccinated mice as described previously^17^. The purified viral particles (WT JEV or JEV-ΔNS1) dissolved in lysis buffer (10% glycerin and 1% Triton X-100 in deionized water) were diluted with coating buffer (0.085 M sodium bicarbonate and 0.015 M sodium carbonate, pH9.5) to 100 ng/ml. One hundred microliters of diluted volumes (10 ng) were added to each well in a 96-well plate. The coated 96-well plates were then blocked with PBS containing 5% skimmed milk. The fourfold serial dilutions (starting at a 1:50 dilution) of heat-inactivated sera from vaccinated mice were added to the coated plates, followed by incubation with the HRP-conjugated goat anti-mouse IgG secondary antibody, and visualized using a two-component 3,3′,5,5′-tetramethylbenzidine (TMB) color development kit (Beyotime Biotechnology). After the addition of 1 M H_2_SO_4_ stop solution, the optical density at 450 nm was measured using a multimode microplate reader (Varioskan Flash; Thermo Fisher) according to the manufacturer’s instruction. The IgG antibody titers were defined as the highest dilution of sera giving an optical density twice that of the nonimmune sera.

### Plaque reduction neutralization test (PRNT)

The neutralizing activities of sera were assayed by PRNT as described previously^17,48,51^. Briefly, serial twofold dilutions of heat-inactivated serum samples (starting at a 1:20 dilution) were preincubated with WT JEV for 1 h at 37 °C and then added to 12-well plates pre-seeded with BHK-21 cells. The mixture was then removed followed by virus quantification by plaque assay. The neutralizing antibody titers (PRNT_50_) were calculated as the highest serial dilutions producing a 50% reduction in viral plaque numbers compared with the control.

### Statistical analysis

The unpaired *t* test or one-way ANOVA was used to determine whether there were significant differences (*p* < 0.05) in all experiments. Kaplan−Meier survival curves were analyzed by the log-rank test. The statistical analyses were performed using nonparametric test in GraphPad Prism software 5.0.

### Reporting summary

Further information on research design is available in the Nature Research Reporting Summary linked to this article.

## Supplementary information

Supplementary Information

Reporting Summary

## Data Availability

All data to understand and assess the conclusions of this study are available in this published article. The raw data that support the findings of this study are available from the corresponding author upon reasonable request.
