# Supplementary material for: A replication-defective Japanese encephalitis virus (JEV) vaccine candidate with NS1 deletion confers dual protection against JEV and West Nile virus in mice
Source: NPJ Vaccines. 2020 Aug 5;5:73. doi: 10.1038/s41541-020-00220-4 (PMC7406499; doi:10.1038/s41541-020-00220-4)
Supplement: Supplementary file 1 — Supplementary Information [file 41541_2020_220_MOESM1_ESM.pdf]

Supplementary information

Supplementary Figure 1

a

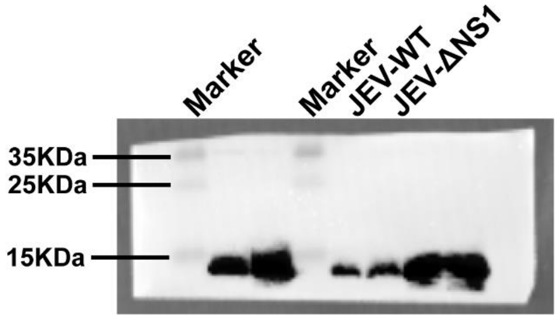

b

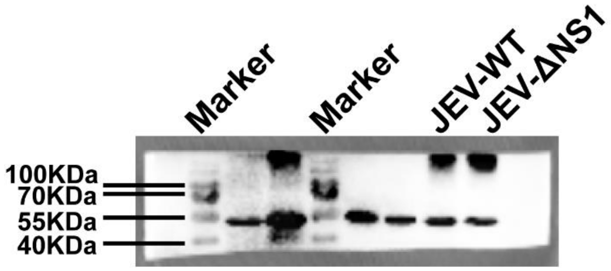

Supplementary Figure 1. Raw images of Fig. 2b. The uncropped and unprocessed blots of capsid (a) and E (b). The molecular weight markers were displayed on the left, the lanes of capsid and E (JEV-WT and JEV-ΔNS1) we displayed in Fig. 2b were marked. The other lanes within the images were constructed for other purposes.

Supplementary Figure 2

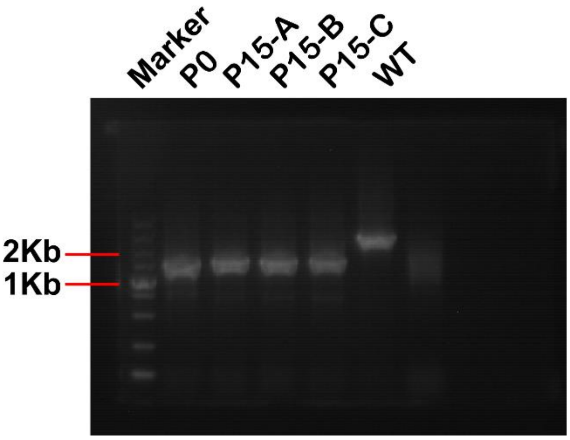

Supplementary Figure 2. Raw images of Fig. 3b. The uncropped and unprocessed gel of Fig. 3b. The fragment length markers were displayed on the left, the lanes we displayed in Fig. 3b were marked.
