# Supplementary material for: A replication-defective Japanese encephalitis virus (JEV) vaccine candidate with NS1 deletion confers dual protection against JEV and West Nile virus in mice
Source: NPJ Vaccines. 2020 Aug 5;5:73. doi: 10.1038/s41541-020-00220-4 (PMC7406499; doi:10.1038/s41541-020-00220-4)
Supplement: Supplementary file 2 — Reporting Summary [file 41541_2020_220_MOESM2_ESM.pdf]

nature research

Corresponding author(s): NPJVACCINES-00649R

Last updated by author(s): Jul 1, 2020

Reporting Summary

Nature Research wishes to improve the reproducibility of the work that we publish. This form provides structure for consistency and transparency in reporting. For further information on Nature Research policies, see our Editorial Policies and the Editorial Policy Checklist.

Statistics

For all statistical analyses, confirm that the following items are present in the figure legend, table legend, main text, or Methods section.

n/a	Confirmed	
☐	☒	The exact sample size (n) for each experimental group/condition, given as a discrete number and unit of measurement
☐	☒	A statement on whether measurements were taken from distinct samples or whether the same sample was measured repeatedly
☐	☒	The statistical test(s) used AND whether they are one- or two-sided<br>*Only common tests should be described solely by name; describe more complex techniques in the Methods section.*
☐	☒	A description of all covariates tested
☐	☒	A description of any assumptions or corrections, such as tests of normality and adjustment for multiple comparisons
☐	☒	A full description of the statistical parameters including central tendency (e.g. means) or other basic estimates (e.g. regression coefficient) AND variation (e.g. standard deviation) or associated estimates of uncertainty (e.g. confidence intervals)
☐	☒	For null hypothesis testing, the test statistic (e.g. F, t, r) with confidence intervals, effect sizes, degrees of freedom and P value noted<br>*Give P values as exact values whenever suitable.*
☒	☐	For Bayesian analysis, information on the choice of priors and Markov chain Monte Carlo settings
☒	☐	For hierarchical and complex designs, identification of the appropriate level for tests and full reporting of outcomes
☒	☐	Estimates of effect sizes (e.g. Cohen's d, Pearson's r), indicating how they were calculated

Our web collection on statistics for biologists contains articles on many of the points above.

Software and code

Policy information about availability of computer code

Data collection	*Provide a description of all commercial, open source and custom code used to collect the data in this study, specifying the version used OR state that no software was used.*
Data analysis	*Provide a description of all commercial, open source and custom code used to analyse the data in this study, specifying the version used OR state that no software was used.*

For manuscripts utilizing custom algorithms or software that are central to the research but not yet described in published literature, software must be made available to editors and reviewers. We strongly encourage code deposition in a community repository (e.g. GitHub). See the Nature Research guidelines for submitting code & software for further information.

Data

Policy information about availability of data

All manuscripts must include a data availability statement. This statement should provide the following information, where applicable:

- Accession codes, unique identifiers, or web links for publicly available datasets
- A list of figures that have associated raw data
- A description of any restrictions on data availability

The authors declare that the data supporting the findings of this study are available within the paper.

Field-specific reporting

Please select the one below that is the best fit for your research. If you are not sure, read the appropriate sections before making your selection.

☒ Life sciences ☐ Behavioural & social sciences ☐ Ecological, evolutionary & environmental sciences

For a reference copy of the document with all sections, see nature.com/documents/nr-reporting-summary-flat.pdf

Life sciences study design

All studies must disclose on these points even when the disclosure is negative.

Sample size	For neuroinvasiveness and neurovirulence tests, 5 mice were included in each group. For protection of JEV-ΔNS1 in mice against JEV challenge, 10 mice were included in each group. For protective efficacy of one-shot immunization with JEV-ΔNS1 in C57BL/6 mice, 6 mice were included in each group. For cross-protection against WNV with one-shot low-dose immunization of JEV-ΔNS1, 10 mice were included in PBS- and 3×107 IU of JEV-ΔNS1-immunized groups; 5 mice were included in other groups.
Data exclusions	No data were excluded.
Replication	For safety and protective efficacy assays, two experiments were performed independently and the reproducibility was successful.
Randomization	All mice were allocated into different groups randomly.
Blinding	The investigators were blinded to group allocation during data collection and analysis.

Reporting for specific materials, systems and methods

We require information from authors about some types of materials, experimental systems and methods used in many studies. Here, indicate whether each material, system or method listed is relevant to your study. If you are not sure if a list item applies to your research, read the appropriate section before selecting a response.

Materials & experimental systems

n/a	Involved in the study	
☐	☒	Antibodies
☐	☒	Eukaryotic cell lines
☒	☐	Palaeontology and archaeology
☐	☒	Animals and other organisms
☒	☐	Human research participants
☒	☐	Clinical data
☒	☐	Dual use research of concern

Methods

n/a	Involved in the study	
☒	☐	ChIP-seq
☒	☐	Flow cytometry
☒	☐	MRI-based neuroimaging

Antibodies

Antibodies used	Monoclonal antibody 4G2 against the flavivirus envelope protein; Anti-JEV capsid polyclonal antibody; Fluorescein isothiocyanate (FITC)-conjugated goat anti-mouse IgG and horseradish peroxidase (HRP)-conjugated goat anti-mouse/rabbit IgG secondary antibodies.
Validation	Monoclonal antibody 4G2 against the flavivirus envelope protein was kindly provided by Cheng-Feng Qin (Beijing Institute of Microbiology and Epidemiology, Beijing, China). Anti-JEV capsid polyclonal antibody was purchased from Abcam (China). Fluorescein isothiocyanate (FITC)-conjugated goat anti-mouse IgG and horseradish peroxidase (HRP)-conjugated goat anti-mouse/rabbit IgG secondary antibodies were purchased from Proteintech (China).

Eukaryotic cell lines

Policy information about cell lines

Cell line source(s)	BHK-21 cells (ATCC® CCL-10™) were used in this study.
Authentication	The BHK-21 cells were from ATCC.
Mycoplasma contamination	All cell lines tested negative for mycoplasma contamination.
Commonly misidentified lines (See ICLAC register)	No commonly misidentified lines were used in this study.

Animals and other organisms

Policy information about studies involving animals; ARRIVE guidelines recommended for reporting animal research

Laboratory animals	Four-week-old C57BL/6 female mice and three-week-old ICR female mice were used in this study.
Wild animals	No wild animals were used in this study.
Field-collected samples	No field-collected samples were used in this study.
Ethics oversight	All mice infection experiments related to JEV were performed at an animal biosafety level 2 (ABSL-2) facility in Wuhan Institute of Virology under a protocol approved by the Laboratory Animal Ethics Committee of Wuhan Institute of Virology, CAS (Permit number: WIVA26201902). All mice infection experiments related to WNV were performed at an animal biosafety level 3 (ABSL-3) facility in Wuhan Institute of Virology under a protocol approved by the Laboratory Animal Ethics Committee of Wuhan Institute of Virology, CAS (Permit number: WIVA26201801).

Note that full information on the approval of the study protocol must also be provided in the manuscript.
